# Fluorescence Properties of a Novel Isoquinoline Derivative Tested in an Invertebrate Chordate, *Ciona intestinalis*

**DOI:** 10.1002/cbic.202100058

**Published:** 2021-05-04

**Authors:** Silvia Mercurio, Lisa Moni, Giorgio Scarì, Raoul Manenti, Renata Riva, Roberta Pennati

**Affiliations:** ^1^ Department of Environmental Science and Policy Università degli Studi di Milano via Celoria 10 20133 Milano Italy; ^2^ Department of Chemistry and Industrial Chemistry Università degli Studi di Genova via Dodecaneso 31 16146 Genova Italy; ^3^ Department of Biosciences Università degli Studi di Milano via Celoria 26 20133 Milano Italy

**Keywords:** ascidian, bioassay, fluorescent molecule, nuclear dye, swimming behaviour

## Abstract

3‐Hydroxyisoquinolines (ISOs) and their tautomeric isoquinolin‐3‐ones are heterocycles with attractive biological properties. Here we reported the revisited synthesis of a highly functionalized ISO that showed blue fluorescence and the characterization of its biological properties in an invertebrate animal model, the ascidian *Ciona intestinalis*. Larvae exposed to ISO at concentrations higher than 1 μM showed an intense fluorescence localized in the cell nuclei of all tissues. Moreover, exposure to ISO interfered with larval ability to swim; this neuromuscular effect was reversible. Overall, these results suggested that ISOs can have promising applications as novel fluorescent dyes of the cell nuclei.

## Graphical abstract

**The synthesis** of a fluorescent 3‐hydroxyisoquinoline derivative (**ISO‐1**) has been revised, increasing the amount to gram scale. Its properties were tested in an invertebrate model, the ascidian *Ciona intestinalis*. **ISO‐1** marks the nuclei in a reversible way and immobilizes larvae, suggesting neuroactive properties.

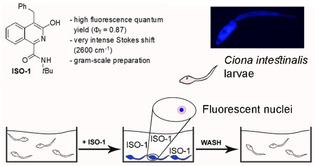

## Introduction

**Scheme 1:**
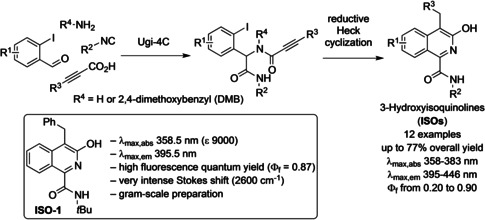
Synthetic strategy for the preparation of a library of substituted 3‐hydroxyisoquinolines and structure and photophysical properties of compound **ISO‐1**.

3‐Hydroxyisoquinolines (ISOs) and their tautomeric isoquinolin‐3‐ones are rather unexplored heterocycles, even if they exhibit attractive biological activities. They have proven to strongly inhibit human 11β‐hydroxydehydrogenase 1 (11β‐HSD1), a privileged target for diabetes and/or metabolic syndrome.^[1]^ ISO derivatives are also potent renal vasodilators^[2]^ and inhibitors against *Plasmodium falciparum* cysteine protease falcipain‐2.^[3]^ Moreover, isoquinoline alkaloids have been isolated in members of the *papaveraceae* family and they showed various biological properties including acetylcholinesterase inhibitory effects, anti‐proliferative activities, antiviral activities and antiplasmodial activities.^[4]^ Recently, we have published a very fast and efficient synthesis of a series of highly functionalized ISOs, heterocyclic structures with excellent properties as blue‐fluorescence emitters and remarkably high fluorescence quantum yields. Our approach consists in a two‐step procedure where a four component Ugi reaction is followed by a palladium‐catalysed reductive Heck cyclization.^[5]^ Actually, ISOs have showed remarkable deep‐blue fluorescence under UV excitation, displaying absorption maxima in the range from 358 and 383 nm, and emission maxima in the range from 395 to 446 nm. The fluorescence efficiency was strongly affected by the substitution of the isoquinoline core as the quantum yields determined with anthracene as a relative standard (Φ_f_=0.36) fall in the range of 0.20 to 0.90 (Scheme 1). The promising photophysical properties of ISOs prompted us to investigate their potential use as fluorescent sensors in different biological applications.

Herein, we describe the revisited synthesis of **ISO‐1**, chosen as model compound, and explore its biological properties in invertebrate animal model, the ascidian *Ciona intestinalis*.

Ascidians are marine organisms closely related to vertebrates.[^6^, ^7^] They have been successfully used to assess the effects of several chemicals on animal development and behaviour.[^8^, ^9^] Adults are sessile filter‐feeding animals that develop through a free‐swimming larva. Larvae display the typical chordate body plan consisting of a trunk that houses the anterior part of the dorsal central nervous system with the sensory organs, and a muscular tail used for locomotion.^[10]^ *Ciona intestinalis*, one of the most studied ascidian species, has been established as a reliable model animal for toxicological tests.[^11^, ^12^, ^13^] By in vitro fertilization, it is possible to obtain hundreds of synchronous embryos that complete their development in almost 18 hours at 18 °C^[14]^ thus allowing extremely quick and powerful toxicological and behavioural tests. Moreover, *Ciona intestinalis* larvae have a stereotyped behaviour making them a suitable model to test the effects of chemicals on their neuromuscular system. Soon after hatching, larvae show a positive phototropism and swim by lateral bending of their tail towards the surface. After few hours, they turn into negative phototropic and start to move toward the substrate searching for an appropriate site to which they adhere by means of three adhesive papillae.[^15^, ^16^] In addition to their numerous practical advantages, ascidians phylogenetic position makes these organisms particularly suitable for testing chemicals. Information about chemical effects in both invertebrates and vertebrates can indeed be extrapolated by results obtained in these animals.

## Results and Discussion

### Gram‐scale synthesis of ISO‐1

**Scheme 2:**
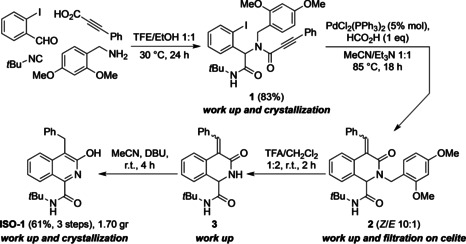
Gram‐scale synthesis of **ISO‐1**.

Previous approach to synthesize the highly functionalized 3‐hydroxyisoquinolines have only resulted in milligram quantities, whereas gram quantities would be required for *in vivo* studies. With this aim, a gram‐scale synthesis of **ISO‐1** has been realized. The synthetic strategy has been depicted in Scheme 2. The Ugi reaction has been carried out in trifluoroethanol (TFE)/ethanol as solvent, employing 2,4‐dimethoxybenzylamine as an ammonia surrogate, obtaining **1** in high yield and purity, after a simple work up to remove the excess of acid and a final crystallization. For the subsequent intramolecular reductive Heck reaction, a stoichiometric amount of formic acid is found to be crucial in order to promote the complete reductive elimination of palladium affording intermediate **2** as a mixture of *Z*/*E* diastereoisomers. After filtration on celite to remove palladium‐related impurities, the mixture was treated with trifluoroacetic acid (TFA) to give **3**, then with 1,8‐diazabicyclo[5.4.0]undec‐7‐ene (DBU) in MeCN to promote the aromatization. Apart from four very fast work‐up and two crystallizations, the sequence was performed without isolating any intermediate, and especially without any chromatographic purification (Scheme 2).

### ISO‐1 effects on development

**Figure 1:**
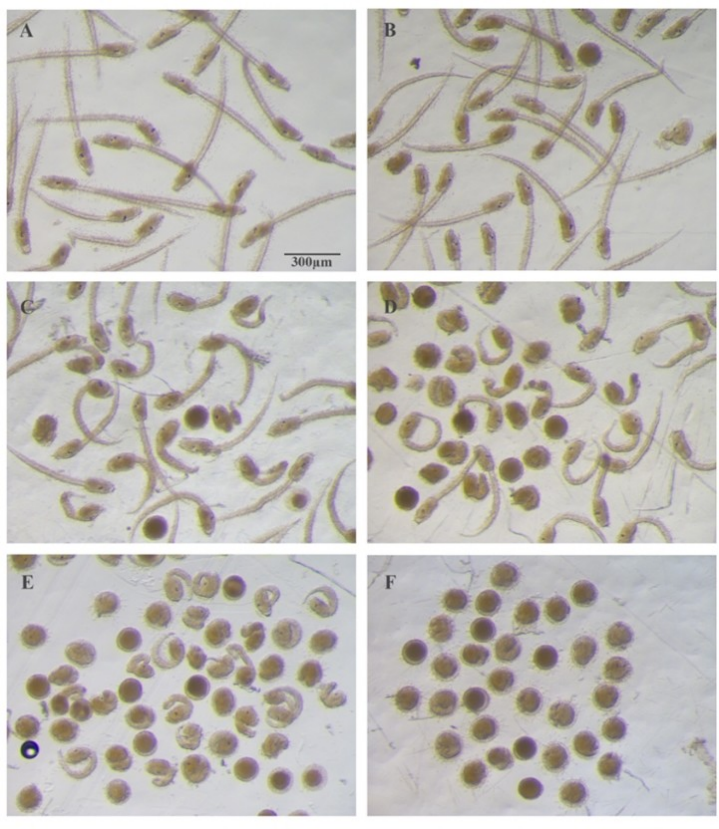
Phenotypes of larvae developed from embryos exposed to different concentrations of **ISO‐1**. A) Control larvae exposed to DMSO. B) Larvae exposed to 1 μM **ISO‐1** showing a normal phenotype. C) Larvae exposed to 5 μM **ISO‐1**. D) Larvae exposed to 10 μM **ISO‐1**. E) Larvae exposed to 20 μM **ISO‐1**. F) Larvae exposed to 25 μM **ISO‐1**.

**Figure 2:**
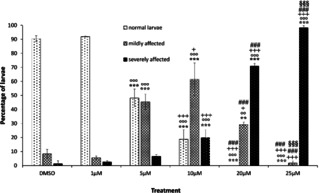
Graph showing the incidence of different phenotypes (normal, mildly, severely affected) in larvae developed from embryos treated with different concentrations of **ISO‐1**. Mean values of three replicates and standard errors are indicated. Legend of symbols: *= differences from control, °=different from 1 μM; +=different from 5 μM; #=different from 10 μM; §=different from 20 μM. The repetition of each symbol indicates the level of significance according to ANOVA significance codes: p<0.001 ***; p<0.01 **; p<0.05 *.

Control larvae as well as those developed in Artificial Sea Water‐HEPES (ASWH) plus 0.02 % DMSO displayed the typical larval morphology. They were characterized by an elongated trunk with two pigmented sensory organs, clearly visible in the sensory vesicle, and a straight long tail (Figure 1A). **ISO‐1** concentration significantly affected *Ciona intestinalis* development in terms of number of normal developed individuals (ANOVA: F=351.72; P=<0.001), number of mildly affected individuals (ANOVA: F=57.64; P=<0.001), and number of severely damaged individuals (ANOVA: F=641.56; P=<0.001). Larvae exposed to the lowest tested concentration of **ISO‐1** (1 μM; Figure 1B) were similar to the control ones and the incidence of adverse effects (either mildly or severely affected larvae) was not significantly different from controls (Tukey's post hoc test: P>0.05). Exposure to 5 μM **ISO‐1** caused a statistically significant increase of mild malformations (Tukey's post hoc test: P<0.001; Figure 2): almost 50 % of larvae showed short and bent tail, round trunk with a small sensory vesicle with close pigmented cells (Figure 1C).

Exposure to higher concentrations caused a significant increase of severe malformations (Tukey's post hoc test: P<0.001) characterized by a curled tail, round trunk, small sensory vesicle with no pigmented organs (Figure 1D).

These kinds of malformations have been reported after exposure to different chemicals and are considered to be due to toxic effects caused by a pleiotropic action of the molecule.^[8]^ Twenty per cent of larvae treated with 10 μM **ISO‐1** showed severe malformations. This percentage gradually increased with **ISO‐1** doses reaching 100 % in larvae exposed to 25 μM **ISO‐1** (Figure 2). Embryos exposed to 50 μM **ISO‐1** died at early developmental stages (data not showed).

**Figure 3:**
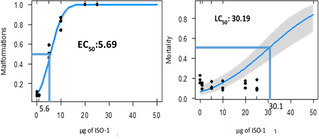
EC_50_ and LC_50_ predicted values calculated with Probit analysis.

Performing Probit analysis, we determined **ISO‐1** toxicological features during *Ciona intestinalis* embryogenesis (Figure 3). The concentration of **ISO‐1** that caused adverse effects in 50 % of exposed sample (median effective concentration, EC_50_) was 5.69 μM. The median lethal concentration (LC_50_) predicted by the analysis was 30.19 μM.

### Fluorescence properties of ISO‐1

**Figure 4:**
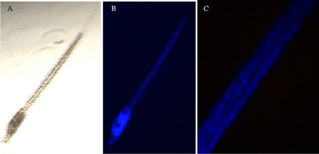
A living larva exposed to 1 μM ISO for 15 minutes. A) Larva observed at the light microscope. B) The same larva of A observed with a UV light and a DAPI filter. The fluorescence signal is concentrated in the trunk. C) Higher magnification of the tail of B shows the signal in the nuclei.

Hatched larvae developed in ASWH were exposed to different concentrations of **ISO‐1** for 1 hour. After this time, a fluorescence signal could be observed using a microscope equipped with a UV lamp and a DAPI filter (band pass 352 to 477 nm). The intensity of the signal appeared proportional to the tested concentrations and was detectable only in larvae exposed to concentrations higher than 1 μM. At high magnification, fluorescent signal was clearly localized in the nuclei of the cells (Figure 4).

### ISO‐1 effects on larvae behaviour

**Figure 5:**
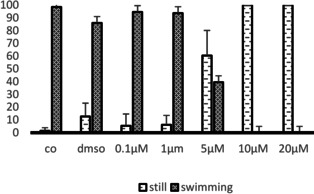
Graph showing the percentage of swimming and resting larvae after an exposure of 15 minutes to different concentrations of **ISO‐1**.

Exposure to **ISO‐1** interfered with larval ability to swim. After 1 hour, 90 % of larvae exposed to 1 μM **ISO‐1** could swim, a percentage comparable to that of larvae exposed to DMSO alone. On the contrary, only 40 % of larvae exposed to 5 μM **ISO‐1** showed a normal swimming behaviour (Figure 5). Concentrations of **ISO‐1** higher than 5 μM blocked tail movements resulting in an increasing number of larvae that appeared resting on the bottom of Petri dishes (Figure 5).

The number of resting larvae was significantly higher at concentrations of 5 μM, 10 μM and 20 μM than at control conditions (Table 1).

**Table 1:** Effects of **ISO‐1** exposure on the number of *Ciona intestinalis* larvae that were not able to swim.

Treatment^[a]^	Estimate	SE	t	P
1 μM **ISO‐1**	8.99E−15	3.89E+00	0	1
5 μM **ISO‐1**	2.27E+01	3.89E+00	5.825	**<0.001**
10 μM **ISO‐1**	4.43E+01	3.89E+00	11.393	**<0.001**
20 μM **ISO‐1**	3.90E+01	3.89E+00	10.022	**<0.001**
Control	−1.33E+0	3.89E+00	−0.343	0.737
DMSO	2.00E+00	3.89E+00	0.514	0.615

[a] The table reports the control‐treatment contrasts; significant effects are in bold.

**Figure 6:**
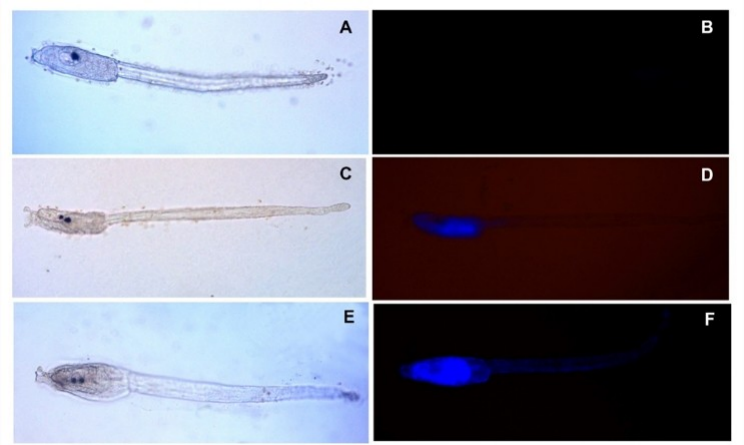
Larvae rinsed in ASWH after exposure to **ISO‐1** observed at light (A, C, E) and fluorescent microscope (B, D, F). A, B) Larvae exposed to 1 μM **ISO‐1**, the fluorescent signal disappeared completely. C, D) A faint signal was still present in larvae exposed to 5 μM **ISO‐1**. E, F) The fluorescent signal persisted in larvae exposed to 10 μM **ISO‐1**.

These effects were partially reversible. In fact, after two short rinses in ASWH, all larvae exposed to 5 μM **ISO‐1** were able to swim again (Table 2) even if they maintained a faint fluorescence visible mainly in the trunk, where the cells are smaller and densely packed (Figure 6).

**Table 2:** T Effects of **ISO‐1** on swimming behaviour and fluorescent signal during the exposure and after rinsing.

	During exposure		After exposure	
Treatment	Swimming	Fluorescence	Swimming	Fluorescence
1 μM **ISO‐1**	+	+	+	–
5 μM **ISO‐1**	–	+	+	+
10 μM **ISO‐1**	–	+	–	+
20 μM **ISO‐1**	–	+	–	+
DMSO	+	–	+	–

### Cell viability assay

The experiment of cells viability revealed a viability rate of 96.05 % in control treatment, 89.36 % in treatment with 0.01 μM **ISO‐1**, 89.36 % in treatment with 0.1 μM **ISO‐1** and 83.33 % in treatment with 1 μM **ISO‐1**. Fisher's exact test showed that **ISO‐1** treatments determined always a significant difference in the rate of viable and dead cells by respect to control treatment (P≥0.01).

In the present work, we explored the biological properties of 3‐Hydroxyisoquinoline derivative **ISO‐1** in the animal model *Ciona intestinalis*, documenting its potentialities in different aspects of scientific research.

Using an established ascidian‐based approach, we identified **ISO‐1** ability to stain specifically cellular nuclei. This peculiar feature, coupled with our revisited synthesis, which increases the amount of **ISO‐1** to gram scale, makes **ISO‐1** an effective vital fluorescent dye with numerous possible applications in *in vivo* tests. Based on our embryotoxicity analysis, **ISO‐1** affected ascidian development at concentrations higher than 5 μM, with a predicted median lethal concentration of 30.19 μM. These concentrations are far from the **ISO‐1** dose necessary to induce fluorescence in multicellular living organisms such as ascidians larvae. In fact, the lowest tested concentration that elicited a fluorescent signal was 1 μM. The signal was strong enough for being detected with a simple UV lamp and a DAPI filter. Indeed, even low concentrations of **ISO‐1** can affect the viability of cultured HEK‐293 cells, which are more sensitive to the action of drugs than multicellular organisms. Fluorescence localized mainly in cell nuclei and larvae exposed to this concentration appeared normal and healthy, as well as able to swim. These promising results raised the possibility to use **ISO‐1** as a vital dye to mark nuclei in a reversible way as we demonstrated that **ISO‐1** labelling could be eliminated by rapid wash in sea water. All these features strongly suggested a high affinity of **ISO‐1** for the nuclei. Future investigations will be focused on clarifying **ISO‐1** binding affinity for nucleic acids and its interactions and/or possible damages to DNA after prolonged exposure.

Another fascinating property of **ISO‐1** is related to its effects on larval behaviour. We found that **ISO‐1**, even at low concentrations such as 5 μM, could reversibly impair larvae swimming abilities. **ISO‐1** immobilizing effects were transient, lasting as long as the larvae were maintained in the **ISO‐1** solution. Ascidian larvae movements are determined by lateral contractions of their tail muscles. Muscles contractions are under the control of the central nervous system,^[19]^ and particularly, are driven by five pairs of GABAergic motor neurons that project axons to the muscle fibres in the tail.[^10^, ^20^] Even if the mechanism by which **ISO‐1** interfered with larvae swimming behaviour remains unclear, we can hypothesize that **ISO‐1** has neuroactive properties similarly to those displayed by other isoquinoline derivatives.^[21]^ For example, papaverine is a well‐known isoquinoline derivative that has antispasmodic effects on smooth muscles. It exerts its activity by inhibiting oxygen uptake of mitochondria oxidizing glutamate under phosphorylative conditions.^[22]^ Drotaverine, is an isoquinoline derivative structurally related to papaverine. It inhibits phosphodiesterases hydrolysing cAMP, thereby increasing cAMP concentration, decreasing Ca^2+^ cellular uptake and changing its distribution among the cells. It is used as an antispasmodic drug, with no anticholinergic effects.

## Conclusion

Even though the molecular mechanisms by which **ISO‐1** could exert its biological properties are still to be clearly determined, this novel molecule have showed attractive and promising features worth to be further analysed. The results obtained in ascidian model underlined the potentialities of this chemical, paving the way for future investigations on **ISO‐1** biological interactions, and its applications as fluorescent biosensor.

## Experimental Section

### Chemicals and animals

Isochinoline (**ISO‐1**) was synthetized according to our procedure.^[5]^ Gram‐scale synthesis of Ugi product: a solution of 2‐iodobenzaldehyde (2.85 g, 12.28 mmol) in TFE/EtOH (1 : 1, 26 mL) was treated at r.t. with 2,4‐dimethoxybenzylamine (1.94 mL, 12.89 mmol). After 30 min the solution was treated at r.t. with phenylpropiolic acid (1.88 g, 12.89 mmol) and *tert*‐butylisocyanide (1.46 mL, 12.89 mmol) and the mixture was stirred at 30 °C. After 24 h, the solvent was removed by evaporation. The crude was treated with saturated aqueous NaHCO_3_ (60 mL) and extracted three times with CH_2_Cl_2_ (150+50+50 mL). The combined organic phases were dried (Na_2_SO_4_), filtered and concentrated. The residue was purified by crystallization from Et_2_O to give the Ugi product (6.19 g, 83 %) as a white solid. The analytical data of this compound are in agreement with those already reported by us. Gram‐scale synthesis of 3‐hydroxyisoquinoline: a solution of Ugi product (5.19 g, 8.50 mmol) in MeCN/Et_3_N (1 : 1, 80 mL) under argon atmosphere, was treated with PdCl_2_(PPh_3_)_2_ (316 mg, 0.42 mmol) and formic acid (3.20 mL, 8.50 mmol) at 85 °C for 18 h. After this time, the solvent was evaporated and the crude was diluted with saturated aqueous NH_4_Cl (80 mL) and extracted three times with CH_2_Cl_2_ (150+150+50 mL). The combined organic phases were filtered through a celite cake, washing thoroughly with CH_2_Cl_2_, dried (Na_2_SO_4_), filtered and concentrated. The residue was diluted with CH_2_Cl_2_ (40 mL) and treated with TFA (20 mL) at room temperature for 2 h. Then, TFA was evaporated and the crude was diluted with saturated aqueous NaHCO_3_ (100 mL), extracted three times with CH_2_Cl_2_ (150+80+80 mL), dried (Na_2_SO_4_), filtered and concentrated. The residue was directly dissolved in dry MeCN (30 mL) and DBU (2.50 mL, 17.00 mmol) was added. The reaction was stirred at r.t. for 4 h, then diluted with CH_2_Cl_2_ and washed with saturated aqueous NH_4_Cl. The combined organic phases were filtered through a celite cake, washing thoroughly with CH_2_Cl_2_, dried (Na_2_SO_4_), filtered and concentrated. The residue was purified by crystallization from CH_2_Cl_2_/Et_2_O to give the **ISO‐1** (1.72 g, 61 %) as a yellow solid. The analytical data of this compound are in agreement with those already reported by us.^[5]^ HPLC (see SI) showed a purity of 99.6 %.

Stock solution of 100 mM **ISO‐1** was made in dimethyl sulfoxide (DMSO) and then diluted in filtered artificial sea water with 1 M HEPES pH 8.0 (ASWH) to reach the final test concentrations.

*Ciona intestinalis* adults were collected from natural populations in Roscoff (France) by the fishing service of the Station Biologique de Roscoff, and reared in aquaria equipped with mechanical, chemical and biological filters. Animals were maintained at 16±1 °C and in constant light conditions to avoid gamete release and stimulate their production.^[17]^ Eggs and sperm were obtained by dissecting the gonoducts and cross‐fertilization was performed. All experiments were carried out at 18±1 °C.

### Effects of ISO‐1 exposure on *Ciona intestinalis* development

To test the effect of **ISO‐1** on the development of *Ciona intestinalis*, embryos at two‐cell stage (∼1 hr post fertilization (hpf)^[14]^ were exposed to 1, 5, 10, 20, 25 and 50 μM **ISO‐1** in ASWH. Fresh solutions were prepared every time. Control embryos were maintained in ASWH plus 0.02 % DMSO. For each treatment, ∼100 embryos were transferred into glass Petri dishes (Ø 9 cm) filled with in 50 ml of experimental solution. Each set of treatments were replicated three time with different batches. Embryos were reared at 18 °C in a thermostatic chamber until they reached the swimming larva stage (∼18 hpf), and then fixed in 4 % paraformaldehyde in phosphate buffer saline (PBS) for 1 h for the following analyses.

The morphological effects of **ISO‐1** exposure were evaluated under a dissection microscope. Then, fixed larvae were rinsed in PBS 3 times for 10 minutes, mounted on glass slides and carefully observed at a transmission microscope for detailed analysis.

Control and treated larvae were scored according to their morphology and classified in three groups: normal phenotype; mildly affected larvae showing malformations in the trunk and in the pigmented sensory organs; severely affected larvae displaying short trunk and a curled and/or short tail.

### ISO‐1 effects on larval behaviour

To test the effects of **ISO‐1** on larval behaviour, 30 swimming larvae, 1 hour after hatching, were exposed to different concentrations of **ISO‐1** for 15 min. Then, larvae were observed under an inverted microscope equipped with a UV lamp, a DAPI filter and a DMC5400 camera (Leica). We recorded the larvae for 5 min and scored the number of fluorescent larvae as well as the number of swimming ones. Then, the larvae were rinsed twice in ASWH for 5 min and observed again under the microscope. The number of swimming, resting and fluorescent larvae was annotated.

### Cell viability assay

To test the effects on cell viability of **ISO‐1**, 80 % confluent HEK‐293 cells were exposed to 1, 0.1 and 0.01 μM **ISO‐1** for 15 min. Suspended cells were centrifuged at 9 g and resuspended in 1 ml PBS. 10 μl of this suspension were mixed with 10 μl of trypan blue stain and allowed to stay at room temperature for 3 min. Viable (unstained) and nonviable cells were counted in a hemocytometer and the percentage of viable cells (viability) was calculated. A Fisher exact test was applied to assess the significance of the observed differences.

### Statistical analysis

First, we assessed the effect of **ISO‐1** on *Ciona intestinalis* larvae development. We performed analyses of variance (ANOVA) followed by HSD Tukey's post hoc test considering the effects of the different **ISO‐1** concentrations on both mildly and severely affected larvae. Prior to analyses, we verified the homogeneity and normality of the variances. Subsequently, we performed a Probit analysis following the simple least squares regression method to calculate the median lethal concentration (LC_50_) and the median effective concentration (EC_50_) of **ISO‐1** on *Ciona intestinalis* embryos. To assess the effect of **ISO‐1** on larvae behaviour, we built a linear model (LM) using as dependent variable the number of resting larvae and the **ISO‐1** concentration as fixed factor. We then used control‐treatment contrasts to compare the different exposure conditions against the controls. All the analyses were performed in the R 3.6.3 environment using the packages vegan, visreg and lme4.^[18]^

## Conflict of interest

The authors declare no conflict of interest.

## Supporting information

As a service to our authors and readers, this journal provides supporting information supplied by the authors. Such materials are peer reviewed and may be re‐organized for online delivery, but are not copy‐edited or typeset. Technical support issues arising from supporting information (other than missing files) should be addressed to the authors.

Supplementary
