# Supplementary material for: Fluorescence Properties of a Novel Isoquinoline Derivative Tested in an Invertebrate Chordate, Ciona intestinalis
Source: Chembiochem. 2021 May 4;22(12):2140–5. doi: 10.1002/cbic.202100058 (PMC8251550; doi:10.1002/cbic.202100058)
Supplement: Supplementary file 1 — Supplementary [file CBIC-22-2140-s001.pdf]

ChemBioChem

Supporting Information

Fluorescence Properties of a Novel Isoquinoline Derivative Tested in an Invertebrate Chordate, *Ciona intestinalis*

Silvia Mercurio[+], Lisa Moni[+], Giorgio Scarì, Raoul Manenti, Renata Riva,* and Roberta Pennati*

Copies of HPLC chromatogram of ISO-1

```
Data File C:\HPCHEM\2\DATA\LISA\LMO524_0.D                            Sample Name: LMO-524 solido3

    Colonna C6 fenilica 150 x 3 mm, 3 micron + precolonna +
    inline filter prima della colonna; LMO-524 solido3  (Co
    nc.:100ug/ml CH3CN); flow=0.34ml/min; Vinj 5ul; Temp: 2
    5°C Term.OFF, VWD=220nm;  A= H2O - B=CH3CN, grad. 0min
    A=70%, 20min A=0%

    =====================================================================
    Injection Date  : 9/18/2020 11:44:53 AM
    Sample Name     : LMO-524 solido3                 Location : Vial 2
    Acq. Operator   :  AeVeO
    Acq. Instrument : 1100                          Inj Volume : 5 µl
    Method          : C:\HPCHEM\2\METHODS\MS-ACN.M
    Last changed    : 9/18/2020 11:54:48 AM by  AeVeO
                      (modified after loading)
```

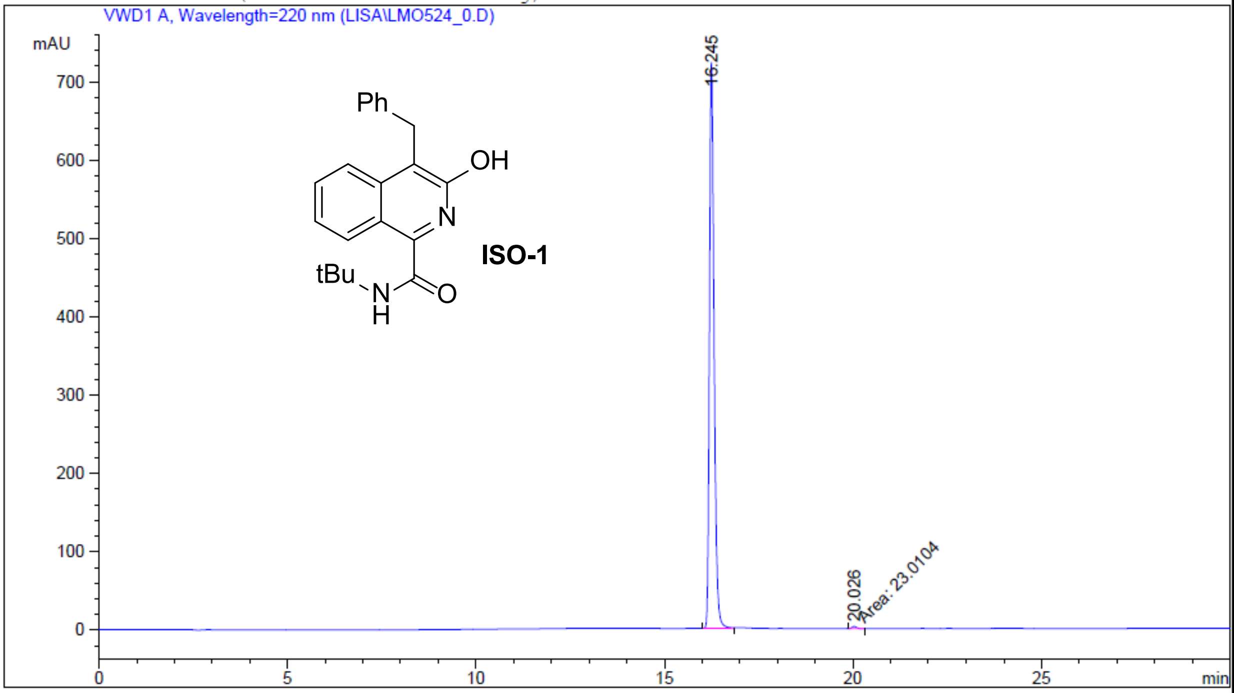


```
    =====================================================================
                         Area Percent Report
    =====================================================================

    Sorted By              :      Signal
    Multiplier             :      1.0000
    Dilution               :      1.0000
    Use Multiplier & Dilution Factor with ISTDs


    Signal 1: VWD1 A, Wavelength=220 nm

    Peak RetTime Type  Width     Area      Height     Area
      #   [min]        [min]  mAU   *s    [mAU   ]     %
    ----|-------|----|-------|----------|----------|--------|
       1  16.245 BB    0.1305 6183.58252  722.32520  99.6293
       2  20.026 MM    0.1379   23.01042    2.78077   0.3707

    Totals :                  6206.59294  725.10597

     Results obtained with enhanced integrator!
    =====================================================================
                              *** End of Report ***
```
